# Intervening to reduce workplace sitting: mediating role of social-cognitive constructs during a cluster randomised controlled trial

**DOI:** 10.1186/s12966-017-0483-1

**Published:** 2017-03-06

**Authors:** Nyssa T. Hadgraft, Elisabeth A. H. Winkler, Genevieve N. Healy, Brigid M. Lynch, Maike Neuhaus, Elizabeth G. Eakin, David W. Dunstan, Neville Owen, Brianna S. Fjeldsoe

**Affiliations:** 1 Physical Activity Laboratory, Baker Heart and Diabetes Institute, Melbourne, VIC 3004 Australia; 2 School of Public Health and Preventive Medicine, Monash University, Melbourne, VIC Australia; 3 The University of Queensland, School of Public Health, Brisbane, QLD Australia; 4 School of Physiotherapy and Exercise Science, Curtin University, Perth, WA Australia; 5 Cancer Council Victoria, Cancer Epidemiology Centre, Melbourne, VIC Australia; 6 Melbourne School of Population & Global Health, The University of Melbourne, Melbourne, VIC Australia; 7 The University of Queensland, School of Medicine, Brisbane, QLD Australia; 8 Mary MacKillop Institute for Health Research, Australian Catholic University, Melbourne, VIC Australia; 9 School of Exercise and Nutrition Sciences, Deakin University, Burwood, VIC Australia; 10 School of Sport Science, Exercise and Health, The University of Western Australia, Perth, WA Australia; 11 Department of Medicine, Monash University, Melbourne, VIC Australia; 12 Swinburne University of Technology, Melbourne, VIC Australia

**Keywords:** Sedentary, Workplace, Intervention, Mediation

## Abstract

**Background:**

The Stand Up Victoria multi-component intervention successfully reduced workplace sitting time in both the short (three months) and long (12 months) term. To further understand how this intervention worked, we aimed to assess the impact of the intervention on four social-cognitive constructs, and examined whether these constructs mediated intervention effects on workplace sitting time at 3 and 12 months post-baseline.

**Methods:**

Two hundred and thirty one office-based workers (14 worksites, single government employer) were randomised to intervention or control conditions by worksite. The intervention comprised organisational, environmental, and individual level elements. Participant characteristics and social-cognitive constructs (perceived behavioural control, barrier self-efficacy, perceived organisational norms and knowledge) were measured through a self-administered online survey at baseline, 3 months and 12 months. Workplace sitting time (min/8 h day) was measured with the activPAL3 device. Single multi-level mediation models were performed for each construct at both time points.

**Results:**

There were significant intervention effects at 3 months on perceived behavioural control, barrier self-efficacy and perceived organisational norms. Effects on perceived organisational norms were not significant at 12 months. Perceived behavioural control significantly mediated intervention effects at 3 months, accounting for a small portion of the total effect (indirect effect: −8.6 min/8 h day, 95% CI: −18.5, −3.6 min; 7.5% of total effect). At 12 months, barrier self-efficacy significantly mediated the intervention effects on workplace sitting time (indirect effect: −10.3 min/8 h day, 95% CI: −27.3, −2.2; 13.9% of total effect). No significant effects were observed for knowledge at either time point.

**Conclusions:**

Strategies that aim to increase workers’ perceived control and self-efficacy over their sitting time may be helpful components of sedentary behaviour interventions in the workplace. However, social-cognitive factors only partially explain variation in workplace sitting reduction. Understanding the importance of other levels of influence (particularly interpersonal and environmental) for initiating and maintaining workplace sedentary behaviour change will be informative for intervention development and refinement.

**Trial registration:**

This study was prospectively registered with the Australian New Zealand Clinical Trials register (ACTRN12611000742976) on 15 July 2011.

**Electronic supplementary material:**

The online version of this article (doi:10.1186/s12966-017-0483-1) contains supplementary material, which is available to authorized users.

## Background

The workplace is a priority setting for initiatives targeting behavioural risk factors for chronic disease [1]. Time spent sitting (sedentary behaviour) is an identified health risk [2, 3] and many adults accumulate large volumes of sitting during their working hours [4–6]. Accordingly, there has been considerable recent attention to the evaluation of interventions to reduce workplace sitting [7, 8]. Despite evidence of intervention efficacy, there has been much less attention to the pathways through which interventions to reduce sitting in the workplace may exert their impact. An ecological approach—targeting physical and social environmental factors, alongside individual-level factors—is considered best practice for workplace health promotion [9] and for interventions aimed at reducing sedentary behaviour [10]. However, there has been limited empirical investigation into how such an approach may lead to successful behavioural change. Understanding the potential role of social-cognitive factors in contributing to behavioural change may provide insight into some of these mechanisms.

Constructs derived from health behaviour theories, such as Social Cognitive Theory [11] and the Theory of Planned Behaviour [12], are often targeted in physical activity interventions [13]. There is fairly consistent evidence suggesting that self-efficacy (confidence in one’s ability to perform a behaviour) is a correlate of physical activity [14] while social support also appears to be important [14, 15]. Fewer studies have explored associations between social-cognitive factors and sedentary behaviour. A relatively modest body of evidence from cross-sectional studies suggests that certain social-cognitive constructs may be correlates of workplace sitting time. For example, a greater level of perceived behavioural control over sitting has been found to be associated with less workplace sitting time [16, 17] and higher levels of standing at work [18], which is consistent with findings from qualitative research [19]. Social norms that reinforce sitting as being the expected or most appropriate workplace behaviour may also lead to higher levels of workplace sitting [17, 20], while there is some evidence to suggest that knowledge about the potential benefits of regularly breaking up sitting positively impacts on this behaviour [21].

With increasing attention to interventions to reduce workplace sitting, understanding how they work – that is whether the constructs targeted to change are actually being impacted, and whether in turn, a change in targeted constructs mediates a change in workplace sitting – is important to inform their continued development and to improve their effectiveness. No previous studies have examined the social-cognitive mediators of multi-component interventions to reduce workplace sitting time.

To address this evidence gap we examined short (3 month) and long (12 month) term changes in social-cognitive constructs (knowledge, barrier self-efficacy, perceived behavioural control, and perceived organisational norms) following a worksite sedentary behaviour intervention, Stand Up Victoria (SUV). In the SUV trial, significant reductions in workplace sitting time were observed in the intervention group relative to the control group of 99.1 min/8 h workday (95% CI −116.3 to −81.8 min/8 h workday) at three months, and 45.4 min/8 h workday (95% CI: −64.6 to −26.2 min/8 h workday) at 12 months [22]. We also examined whether these constructs mediated the significant intervention effects on participants’ workplace sitting time at these two time-points.

## Methods

### Study design and participants

Stand Up Victoria (SUV) was a cluster randomised controlled trial (RCT) of a multi-component workplace intervention aimed at reducing workplace sitting time. Ethics approval was granted by Alfred Health Human Ethics Committee (Melbourne, Australia), with prospective trial registration with the Australian New Zealand Clinical Trials register (ACTRN12611000742976) on 15 July 2011. The trial was conducted in accordance with the CONSORT guidelines for cluster randomised trials (http://www.consort-statement.org/). Further details of participant recruitment [23], study procedures [24], and the main outcomes [22] have previously been published. In brief, participants were 231 government office-based workers recruited from 14 geographically separate worksites from a single employer in Melbourne, Australia between April 2012 and October 2013. Cluster sizes ranged from 5 to 39 participants. A total of 208 (121 intervention and 87 control) and 167 (97 intervention and 70 control) participants completed the 3 month and 12 month follow up assessments respectively. Randomisation to the control or intervention conditions occurred at the worksite level; outcomes and covariates were measured at the individual level. Due to the nature of the intervention, participants and study staff were not blinded to group allocation.

### Intervention

A multi-component intervention, incorporating elements at the organisational level (e.g. tailored management emails), the built/physical environmental level (sit-stand workstations) and the individual level (e.g. health coaching), was delivered to participants in the intervention sites. Individual- and organisational-level strategies were delivered for 3 months, while the workstations were retained for 12 months. The three intervention messages of “Stand Up, Sit Less, Move More” intended to reduce sitting time, particularly prolonged durations of sitting time, through replacement with standing or light intensity (e.g. walking) activities. Control site participants were advised of the aim of the study and continued their usual work practices. Further details of the iterative development of the intervention have been published previously [25]. Briefly, the intervention was informed by Social Cognitive Theory [11], workplace health promotion frameworks including the World Health Organization’s Healthy Workplace Framework [26], and formative research [4, 27, 28]. The final SUV intervention also incorporated a participatory approach that influenced the specific behaviour change techniques adopted [25].

The intervention components (detailed in Table 1) aimed to positively influence four key social-cognitive constructs: perceived behavioural control, barrier self-efficacy, organisational social norms and knowledge. The SUV intervention components had an explicit theoretical and pragmatic basis [25], however, the trial did not aim to comprehensively test a single behavioural theory.

**Table 1:** Description of hypothesised social-cognitive mediators and associated intervention strategies

Hypothesised mediators	Scale description	Response	Targeted intervention strategies	Internal consistency (Cronbach’s alpha)
Perceived behavioural control	Perceived control over sitting less at work. E.g. *It is my choice whether I stand up or sit at my desk while at work”*	Five items; 1–5 Likert scale. Strongly disagree - Strongly agree	- Participant brainstorming session to identify strategies to reduce sitting. - Establishing new workplace policies & practices (e.g. standing meetings, no emails within organisational units) - Installation of height-adjustable workstations - Environmental changes to encourage movement (e.g. signs at lifts prompting use of stairs)	0.72
Barrier self-efficacy	Confidence about overcoming barriers to sitting less at work. E.g. *How confident would you have been that you could have stood up during meetings at work, even though no one else was.*	Nine items; 1–5 Likert scale. Not at all confident - Very confident	- SMART goal setting for use of workstations with health coach - Problem solving with health coach to overcome barriers - Encouraging use of prompts (e.g. stand when telephone rings) - Encouraging use of strategies (e.g. “imails” instead of emails)	0.92
Perceived organisational norms	Perceived organisational/social support for sitting less at work. E.g. *My workplace is committed to supporting staff choices to stand or move more at work*	Eight items; 1–5 Likert scale. Strongly disagree - Strongly agree	- Organisational/upper management support - Team champions acting as role models and spokespersons - Tailored management emails sent from team champions - Establish new workplace policies & practices (e.g. standing meetings, no emails within organisational units)	0.81
Knowledge	Knowledge about the health effects of prolonged sitting. E.g. *Sitting for most of the time at work is bad for my health*	Five items; 1–5 Likert scale. Strongly disagree - Strongly agree	- Information session on the health consequences of excessive sitting - Health coaching - Management emails with information on health effects	0.50

### Data collection and measures

#### Workplace sitting

Onsite assessments were conducted at baseline, 3 and 12 months for both intervention and control groups. These included collection of anthropometric and cardio-metabolic measures and provision of instructions for wearing the activPAL3 activity monitor (PAL Technologies Limited, Glasgow, UK), which was used to measure the primary outcome, workplace sitting time. The activPAL3 is considered accurate and responsive in measuring sitting time [29]. The monitor was waterproofed and attached to participants’ right thigh with a hypoallergenic patch. Participants were asked to wear the monitor for seven days, 24 h/day, following the onsite assessment. A diary was provided for participants to record their working hours, wake and sleep times, and any monitor removal periods. To account for differences in working hours, workplace sitting time was standardised to an 8 h work day.

#### Social-cognitive constructs and covariates

Following each onsite assessment, participants completed a self-administered online questionnaire [30] to collect data on socio-demographic, work-related and health-related factors, and the social-cognitive constructs. Details of the tools used to assess the social-cognitive constructs, including their psychometric properties, are shown in Table 1. As there were no existing measures specific to workplace sitting for these constructs, scales were adapted from the physical activity literature or purposively developed for the study. These measures have previously been pilot-tested [4]. Scores for each construct at the three time points were calculated by averaging responses to individual items and were measured on 5-point Likert scales. The change in participants’ scores on each construct were calculated from i) baseline to three months, and ii) baseline to 12 months; these change scores were used in the mediation analyses.

### Statistical analyses

**Fig. 1:**
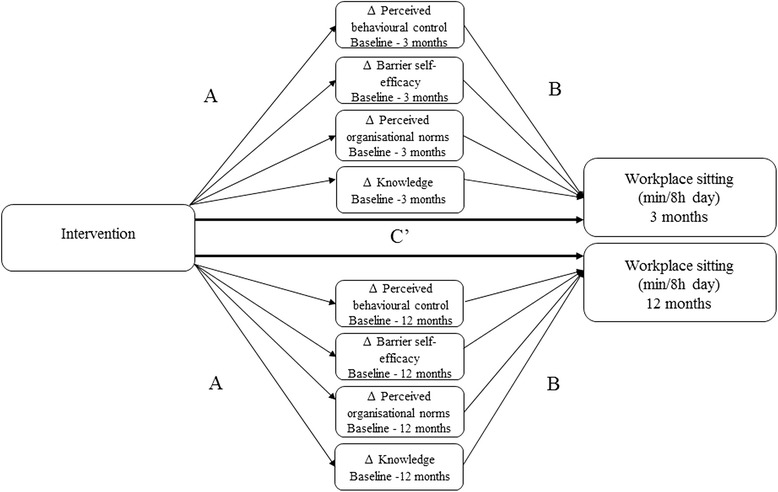
Mediation analysis overview. Path **a** effect of the intervention on the social-cognitive constructs; Path **b** effect of changes in the social-cognitive constructs on workplace sitting time at 3 and 12-months; and Path **c'** direct effects of the intervention on workplace sitting time

Analyses were conducted in STATA v.14 (STATACorp LP) and statistical significance was set at *p* < 0.05. To examine the potential mediating role of changes in social-cognitive constructs on workplace sitting at three, and 12 months, mediational analyses using a completers analysis were performed using STATA’s “ml mediation” package (P.B. Ender, UCLA). This package performs a series of multi-level linear regression analyses to obtain coefficients for: Path A, the effect of the intervention on changes in the social-cognitive constructs; Path B, the relationship between changes in the social-cognitive constructs and changes in workplace sitting; and, Path C’, the direct effects of the intervention on workplace sitting time (see Fig. 1). Mediational effects were calculated by the product of coefficients (a*b) method [31], with bias-corrected confidence intervals determined using cluster bootstrapping with 5000 replications. The coefficients indicate changes in minutes per day of workplace sitting time for each one point increment (on the 5-point Likert scale) for each of the social-cognitive constructs. Separate models were run for each mediator separately (i.e. single mediation) at both time points (i.e. concurrent mediation). All models adjusted for baseline sitting time and potential confounders, and corrected for clustering via a random intercept for worksite. Potential confounders were identified a priori and included in the models if they predicted workplace sitting changes at either 3 or 12 months at *p* < 0.20 using backwards elimination (age and gender were included in all models regardless of significance). Indirect effects are also reported as a percentage of the total intervention effect. Worksite variation was reported from the mixed models in terms of intracluster correlations and the significance of the random intercept for worksite, accounting for confounding variables and intervention/control status. The SUV trial was powered a priori on detecting a minimum difference of interest (MDI) for workplace sitting of 45 min/8 h day between the intervention and control groups with 90% power [22]. Our MDI in the social-cognitive constructs was 0.5, which is equivalent to 50% of participants changing by 1 point on the 5-point Likert scales. Effects less than this were considered “small”.

As a sensitivity analysis, the effect of the intervention on the social-cognitive constructs (Path A) was analysed using intention-to-treat principles [32]. All randomised participants were evaluated using multiple imputation (m = 30 imputations) by chained equations.

## Results

The mean age of the 231 participants at baseline was 45.6 ± 9.4 years. A majority were female (68.4%), Caucasian (79.7%), had post-school education (66.8%) and worked full-time (79.2%). Additional baseline characteristics by worksite have been reported previously [23]. Complete data for the mediation analyses were available for 186 participants at 3 months and 145 participants at 12 months; participants included in the analyses were representative of the sample as a whole with regards to socio-demographic and work characteristics. Additional file 1 shows participant scores for the social-cognitive constructs, including individual items, at baseline, 3 months and 12 months.

### Intervention effects on social-cognitive constructs (Path A)

The effect of the intervention on the social-cognitive constructs is presented in Table 2. At 3 months, there were significant intervention effects on perceived behavioural control, barrier self-efficacy and perceived organisational norms, favouring the intervention group. The effects on perceived behavioural control and barrier self-efficacy met the MDI. Significant differences between the intervention and control groups persisted at 12 months in perceived behavioural control (0.63 points) and barrier self-efficacy (0.54 points). However, at 12 months the effects on perceived organisational norms were small and no longer statistically significant. There was no significant or meaningful intervention effects on knowledge at either time point. As per the main trial outcomes [22], similar results were obtained with multiple imputation and with completers (Additional file 2).

**Table 2:** Effect of the SUV intervention on targeted social-cognitive constructs at 3 and 12 months (Path A)

		Mean change (SE)		Intervention effect (95% CI)^a^	*p*
		Intervention	Control		
Perceived behavioural control	3 months^b^	0.80 (0.09)	0.18 (0.06)	0.67 (0.40, 0.95)	**<0.001**
	12 months^c^	0.82 (0.14)	0.18 (0.10)	0.63 (0.23, 1.03)	**0.002**
Barrier self-efficacy	3 months^b^	0.94 (0.07)	0.11 (0.09)	0.87 (0.58, 1.16)	**<0.001**
	12 months^d^	0.78 (0.11)	0.21 (0.16)	0.54 (0.07, 1.00)	**0.023**
Perceived organisational norms	3 months^b^	0.31 (0.05)	0.07 (0.03)	0.25 (0.10, 0.41)	**0.001**
	12 months^d^	0.22 (0.08)	0.04 (0.07)	0.18 (−0.07, 0.43)	0.163
Knowledge	3 months^b^	0.20 (0.05)	0.02 (0.07)	0.17 (−0.01, 0.36)	0.070
	12 months^c^	0.22 (0.05)	0.19 (0.08)	0.00 (−0.22, 0.23)	0.982

*Note*: For each construct, minimum score = 1 and maximum score =5. SE = standard error, CI = confidence interval. Mean change (SE) are calculated with linearized variance estimation. Significant effects are indicated in bold.

^a^Adjusted for baseline values of the following potential confounders: workplace sitting (min/8 h), age (years), gender (men/women), Caucasian ethnicity (yes/no), current smoking (yes/no), body mass index (log-transformed), AQoL-8D physical superdomain score (log-transformed), AQoL-8D mental superdomain score (log-transformed), TV viewing time (log-transformed), job control category (high/low), weekly headaches (yes/no), musculoskeletal symptoms in the upper extremities (none/does not interfere with activities/interferes with activities)

^b^Intervention: *n* = 110, Control: *n* = 76; ^c^ Intervention: *n* = 89, Control: *n* = 57 ^d^ Intervention: *n* = 88, Control: *n* = 57

The intervention effects on the social-cognitive constructs did not differ significantly by worksite at 3 months (Additional file 3). However, statistically significant worksite effects at 12 month changes were observed for perceived behavioural control (*p* = 0.014, ICC = 0.128, 95% CI: 0.029, 0.421) and perceived organisational norms (*p* = 0.003, ICC = 0.169, 95% CI: 0.042, 0.487).

### Relationships of changes in social-cognitive constructs with changes in workplace sitting (Path B)

Increases in each of the social-cognitive constructs tended to be associated with reductions in workplace sitting (Table 3), although these were only statistically significant for barrier self-efficacy at 12 months (19 min additional reduction in sitting per one point increase on the 5-point scale). Effects of perceived behavioural control at 3 months and knowledge at 3 and 12 months on reductions in sitting were sizeable (approximately 10–15 min per point increase), but did not reach statistical significance.

**Table 3:** Relationships between concurrent changes in social-cognitive constructs with changes in workplace sitting time at 3 and 12 months (Path B)

	Workplace sitting time change		Workplace sitting time change	
	Baseline to 3 months^a^		Baseline to 12 months	
	b (95% CI)^c^	*p*	b (95% CI)^c^	*p*
Perceived behavioural control	−12.74 (−27.08, 1.61)	0.082	−0.44 (−19.72, 18.84)^b^	0.964
Barrier self-efficacy	−5.63 (−16.10, 4.85)	0.292	−19.17 (−32.45, −5.90)^c^	**0.005**
Perceived organisational norms	−5.20 (−24.70, 14.29)	0.601	−0.20 (−23.08, 22.67)^c^	0.986
Knowledge	−11.92 (−27.95, 4.11)	0.145	−13.62 (−33.83, 6.58)^b^	0.186

*Note*: For each construct, minimum score = 1 and maximum score = 5. Significant effects are indicated in bold.

Models adjusted for intervention status and baseline values of the following potential confounders: workplace sitting (min/8 h), age (years), gender (men/women), Caucasian ethnicity (yes/no), current smoking (yes/no), body mass index (log-transformed), AQoL-8D physical superdomain score (log-transformed), AQoL-8D mental superdomain score (log-transformed), TV viewing time (log-transformed), job control category (high/low), weekly headaches (yes/no), musculoskeletal symptoms in the upper extremities (none/does not interfere with activities/interferes with activities)

^a^ *n* = 186; ^b^ *n* = 146 ^c^ *n* = 145

### Mediation effects

Only one social-cognitive construct—perceived behavioural control—significantly mediated the intervention effects for workplace sitting (Table 4) at 3 months, although only a relatively small percentage of the total effect was explained (7.5%). An intervention effect of a 9 min/8 h day reduction co-occurred with each one-point increase in perceived behavioural control (indirect effects); the remaining 106 min/8 h day reduction occurred independently (direct effects). Barrier self-efficacy was a significant mediator of the intervention at 12 months (indirect effect = 10 min/8 h day, 95% CI: −27.26, −2.16), explaining a slightly higher proportion of the total effect (14% mediation). Other indirect effects were all small (<5 min/ 8 h day) and non-significant.

**Table 4:** Mediation of short- and long-term intervention effects on workplace sitting (min/8 h day) by concurrent changes in social-cognitive constructs

Mediators		Direct effect	Indirect effect	Percentage of total intervention effect^d^ mediated
		c' (95% CI)	a*b (95% CI)	
Perceived behavioural control	3 months^a^	−106.34 (−129.35, −75.37)	−8.57 (−18.46, −3.57)	7.46%
	12 months^b^	−74.52 (−114.78, −26.61)	−0.28 (−11.03, 12.99)	0.37%
Barrier self-efficacy	3 months^a^	−110.15 (−135.25, −81.62)	−4.89 (−17.12, 3.82)	4.25%
	12 months^c^	−64.09 (−105.64, −23.41)	−10.34 (−27.26, −2.16)	13.89%
Perceived organisational norms	3 months^a^	−113.74 (−133.79, −85.90)	−1.31 (−5.49, 1.94)	1.14%
	12 months^c^	−74.85 (−112.07, −30.85)	−0.04 (−5.44, 2.86)	0.05%
Knowledge	3 months^a^	−112.85 (−134.65, −85.80)	−2.07 (−8.31, 0.45)	1.80%
	12 months^b^	−73.59 (−109.00, −32.54)	−0.03 (−3.85, 4.15)	0.05%

*Note*: For each construct, minimum score = 1 and maximum score = 5

Models adjusted for baseline values of the following potential confounders: workplace sitting (min/8 h), age (years), gender (men/women), Caucasian ethnicity (yes/no), current smoking (yes/no), body mass index (log-transformed), AQoL-8D physical superdomain score (log-transformed), AQoL-8D mental superdomain score (log-transformed), TV viewing time (log-transformed), job control category (high/low), weekly headaches (yes/no), musculoskeletal symptoms in the upper extremities (none/does not interfere with activities/interferes with activities)

^a^ *n* = 186; ^b^ *n* = 146 ^c^ *n* = 145 ^d^ Total intervention effect (c) comprises direct effect that occurs independently of the mediator (c’) and the indirect effect (ab) that that occurs via the mediator

## Discussion

This multi-component sedentary behaviour intervention significantly improved perceived behavioural control, barrier self-efficacy and perceived organisational norms in the short-term. Knowledge scores increased slightly for intervention group participants at 3 months; however, increases did not significantly exceed control changes. Only changes in perceived behavioural control and barrier self-efficacy reached the minimum difference of interest. Significant intervention effects on perceived behavioural control and barrier self-efficacy were still present at 12 months; effects for perceived organisational norms were no longer statistically significant. In practical terms, this suggests that intervention group participants were more confident that they could overcome barriers to reducing workplace sitting and felt that they had greater levels of control over their activity levels in the workplace, compared with control participants. They also perceived their colleagues and managers to have increased their support of the main intervention messages, particularly in the initial stages of the intervention.

For perceived organisational norms, the non-significant intervention effect at 12 months appeared to be due to a slight drop off in intervention group scores between 3 and 12 months. In our trial, the organisational-level intervention components designed to foster workplace culture largely ceased at 3 months. Future workplace interventions should examine how much additional and/or longer-term support is needed in order to sustain perceived cultural changes related to moving more and sitting less.

Interestingly, there appeared to be a rise in control group participants’ knowledge scores across the intervention, with participants in both the control and intervention groups reporting approximately the same level of knowledge at 12 months. These increases could reflect the significant media attention about sedentary behaviour in Australia during the period in which the trial was conducted [33]. Moreover, as described elsewhere [22], control group participants received the same feedback on their objectively-measured activity levels as did the intervention group participants at three and 12 months. This may also have played a role in fostering their knowledge regarding the detrimental health impacts of high sedentary time. While a recent review found education to be one of the more promising intervention techniques for sedentary behavioural change [34], this may be more relevant for those with a lower starting level of knowledge.

Only one statistically significant mediator of workplace sitting change was identified at 3 months – perceived behavioural control. Consistent with a multi-component intervention (many contributors to the intervention effect), the extent of mediation was small at 10% of the total effect. Previous cross-sectional research [16, 17] has linked higher levels of perceived behavioural control with lower levels of workplace sitting time. In a recent study exploring the utility of the Theory of Planned Behaviour for explaining variation in standing time amongst workers with sit-stand desks, perceived behavioural control was the only theoretical construct found to be significantly related to behaviour [18]. Perceptions of behavioural control may be particularly important for sedentary behaviour in the workplace where there is generally less volitional control than in other settings, such as the home environment [17]. Whether the main driver of changes in perceived behavioural control was the provision of the sit-stand workstations, or a combination of targeted strategies, requires further investigation. Perceived behavioural control was no longer a mediator of workplace sitting time at 12 months. This may suggest that this factor may be more important for the short-term initiation of behavioural change. However, due to the unknown effects of missing data at 12 months (reducing the sample size), caution should be taken in interpreting these results.

Of the other constructs, barrier self-efficacy was a significant mediator at 12 months, explaining nearly 14% of the intervention effect on workplace sitting time. These findings suggest that having the confidence to overcome potential barriers may be important to sustain sitting time reductions in the long-term. Considering that sitting is a highly habitual behaviour [35], participants’ confidence in their ability to stand up in the workplace despite potential barriers may have been particularly important following conclusion of the individual-level support elements (i.e., after 3 months). This is in contrast to two cross-sectional studies (including baseline results of this trial [23]) that failed to find an association between workplace sitting time and self-efficacy [16, 23]. Low levels of self-efficacy amongst participants was suggested as an explanation for the null finding in one of these studies [16]. There is evidence to suggest that high levels of self-efficacy are associated with maintenance of physical activity levels [36, 37]. The potential role of barrier self-efficacy in the maintenance of workplace sitting reduction over time is of interest for future research, as the factors contributing to the sustainability of this behaviour are currently unclear.

Identification of effective elements of multi-component interventions is challenging, but fundamental to advancing knowledge of pathways of successful health behaviour change [38]. This study aimed to understand the mechanisms through which a multi-component intervention contributed to workplace sitting time reductions, by examining the role of social-cognitive influences only. The small effect sizes observed in the mediation analysis suggest that while these social-cognitive factors may play a role in reducing workplace sitting, they are unlikely to have been the main drivers of change. This is in line with a recent review of workplace sedentary behaviour interventions reporting that multi-component interventions, followed by environmental-only interventions, achieved the largest reductions in workplace sitting time, while interventions focusing only on individual-level strategies tended to have a smaller impact [39]. This is further supported by evidence demonstrating the efficacy of a multi-component intervention over a physical environmental change (e.g. sit-stand desk) in isolation [27]. Future studies employing multi-component interventions should also examine other levels of influence, such as interpersonal, environmental and policy factors, and interactions between these levels where possible. The SUV intervention primarily focused on reducing total and prolonged workplace sitting time and was effective in achieving these aims [22]. For future translational research, it may be of interest to consider whether other health risk factors could be addressed alongside the issue of prolonged workplace sitting. For example, workplace policies and support for healthy eating, smoking cessation and active transport could be promoted in conjunction with interventions targeting workplace sitting as part of a comprehensive workplace health promotion program.

This study is the first to examine both short- and longer-term mediation of workplace sitting time reduction. The objective measurement of workplace sitting time and the follow up at two time points are key strengths of this study, as the available evidence on social-cognitive factors associated with sedentary behaviour has largely been limited to cross-sectional studies [16–18]. The main limitations are that these secondary analyses were likely underpowered, particularly at 12 months where over 35% of participants had missing data and were excluded from analyses. We cannot exclude the possibility that significant mediation effects were present at 12 months but were not identified, or that our results were influenced by attrition or participation biases. In addition, the tools used to assess social-cognitive constructs in this study, although previously pilot-tested [4], have not been validated.

## Conclusions

The multi-component Stand Up Victoria trial successfully reduced sitting in the workplace. This study provides insight into some of the mechanisms through which these reductions may have occurred, including examination of short- and long-term mediation effects. Future interventions and programs could consider incorporating behaviour change techniques that aim to foster participants’ level of perceived behavioural control and self-efficacy over their workplace sitting time, alongside modifications to the physical workplace environment. This could include encouraging workers to set goals to increase the time they spend standing or moving, and problem solving barriers to sitting less. Further understanding of the broader array of potential determinants of workplace sitting change will likely be needed to support novel approaches to address this emergent work health and safety issue.

## Additional files

Additional file 1: Control and intervention group scores on social-cognitive construct items at each time-point. (DOCX 20 kb)
Additional file 2: Effect of the SUV intervention on targeted social-cognitive constructs at 3 and 12 months – intention to treat analysis. (DOCX 15 kb)
Additional file 3: Worksite variation (ICCs) in changes in social-cognitive constructs at 3 and 12 months. (DOCX 14 kb)

## Abbreviations

CI: Confidence interval
MDI: Minimum difference of interest
RCT: Randomised controlled trial
SUV: Stand Up Victoria.
