# Supplementary material for: Intervening to reduce workplace sitting: mediating role of social-cognitive constructs during a cluster randomised controlled trial
Source: Int J Behav Nutr Phys Act. 2017 Mar 6;14:27. doi: 10.1186/s12966-017-0483-1 (PMC5340005; doi:10.1186/s12966-017-0483-1)
Supplement: Additional file 1: — Control and intervention group scores on social-cognitive construct items at each time-point. (DOCX 20 kb) [file 12966_2017_483_MOESM1_ESM.docx]

Additional file 1: Control and intervention group scores on social-cognitive construct items at each time-point

|  | **Baseline** | | **3 months** | | **12 months** | |
| --- | --- | --- | --- | --- | --- | --- |
|  | **Control** | **Intervention** | **Control** | **Intervention** | **Control** | **Intervention** |
|  | n=89 | n=133 | n=80 | n=118 | n=64 | n=96^a^ |
|  | mean (sd) | mean (sd) | mean (sd) | mean (sd) | mean (sd) | mean (sd) |
| **Perceived behavioural control** |  |  |  |  |  |  |
| a. It is my choice whether I stand up or sit at my desk while at work | 2.39 (1.24) | 2.50 (1.31) | 2.49 (1.21) | 4.24 (0.79) | 2.44 (1.19) | 3.95 (1.04) |
| b. It is my choice whether I stand up or sit during a meeting with colleagues at work | 2.87 (1.16) | 3.20 (1.17) | 3.23 (1.12) | 4.07 (0.78) | 3.47 (1.02) | 4.02 (0.78) |
| c. It is my choice whether I stand up or sit during a meeting with my supervisor/s at work | 2.78 (1.15) | 2.95 (1.17) | 3.03 (1.15) | 3.69 (1.00) | 3.20 (1.04) | 3.79 (0.91) |
| d. It is my choice whether I walk over to talk to a colleague (*iMail)* or send them an eMail | 3.76 (0.89) | 3.61 (1.05) | 3.81 (0.95) | 3.97 (0.88) | 3.70 (0.98) | 4.00 (0.83) |
| e. It is my choice whether I walk over to talk to a supervisor (*iMail)* or send them an eMail | 3.70 (0.99) | 3.67 (1.00) | 3.83 (0.92) | 3.92 (0.94) | 3.72 (0.93) | 4.01 (0.85) |
| Overall perceived behavioural control score | 3.10 (0.72) | 3.18 (0.80) | 3.28 (0.72) | 3.97 (0.66) | 3.31 (0.74) | 3.95 (0.62) |
| **Barrier self-efficacy** |  |  |  |  |  |  |
| a. Stood up during meetings at work, even though no one else was | 2.47 (1.27) | 2.53 (1.27) | 2.64 (1.38) | 3.36 (1.23) | 2.63 (1.31) | 3.24 (1.21) |
| b. Stood up during meetings at work, even when supervisors were sitting down | 2.27 (1.25) | 2.40 (1.24) | 2.51 (1.34) | 3.19 (1.33) | 2.52 (1.38) | 3.11 (1.21) |
| c. Stood up at your desk at work, even though your colleagues were not | 2.75 (1.36) | 3.09 (1.29) | 2.88 (1.28) | 4.24 (0.86) | 2.89 (1.29) | 3.93 (1.07) |
| d. Stood up at your desk at work, even when you felt tired | 2.69 (1.32) | 2.71 (1.23) | 2.80 (1.27) | 3.60 (1.05) | 2.59 (1.38) | 3.31 (1.14) |
| e. Stood up at your desk at work, even if your footwear was uncomfortable | 2.33 (1.33) | 2.49 (1.15) | 2.43 (1.36) | 3.28 (1.21) | 2.23 (1.25) | 3.07 (1.30) |
| f.  Stood up at your desk at work, even though you were really busy at work | 2.28 (1.39) | 2.53 (1.28) | 2.44 (1.30) | 3.75 (1.04) | 2.42 (1.38) | 3.33 (1.20) |
| g. Stood up at your desk at work, even when your tasks required looking at multiple papers | 2.13 (1.32) | 2.34 (1.22) | 2.29 (1.26) | 3.25 (1.25) | 2.33 (1.37) | 3.04 (1.30) |
| h. Stood up at your desk at work, even when your tasks required talking on the phone | 2.49 (1.34) | 2.69 (1.36) | 2.61 (1.35) | 3.81 (1.11) | 2.55 (1.40) | 3.56 (1.21) |
| i. Walk to talk to a colleague at work instead of emailing them, even though others didn’t | 3.39 (1.26) | 3.45 (1.23) | 3.49 (1.11) | 3.98 (0.96) | 3.34 (1.24) | 3.94 (0.94) |
| Overall barrier self-efficacy score | 2.53 (1.05) | 2.69 (0.97) | 2.68 (1.04) | 3.61 (0.74) | 2.61 (1.08) | 3.39 (0.82) |
| **Perceived organisational norms** |  |  |  |  |  |  |
| a. My workplace is committed to supporting staff health and well-being | 3.60 (0.92) | 3.47 (0.87) | 3.66 (0.88) | 3.64 (0.95) | 3.70 (1.01) | 3.45 (1.02) |
| b.  My workplace is committed to supporting staff choices to stand or move more at work | 2.88 (1.07) | 3.14 (0.97) | 2.93 (1.05) | 3.65 (0.94) | 3.06 (1.06) | 3.22 (1.09) |
| c. My colleagues would not mind if I chose to stand up while working at my desk | 3.71 (0.87) | 3.86 (0.68) | 3.71 (0.84) | 4.20 (0.71) | 3.80 (0.74) | 4.15 (0.68) |
| d. My supervisor/s would not mind if I chose to stand up while working at my desk | 3.57 (0.87) | 3.91 (0.63) | 3.59 (0.93) | 4.24 (0.76) | 3.75 (0.79) | 4.09 (0.82) |
| e. My colleagues would not mind if I chose to stand during a work meeting | 3.54 (0.82) | 3.68 (0.71) | 3.60 (0.80) | 4.01 (0.77) | 3.61 (0.82) | 4.04 (0.77) |
| f. My supervisor/s would not mind if I chose to stand during a work meeting | 3.46 (0.86) | 3.59 (0.74) | 3.51 (0.82) | 3.93 (0.89) | 3.61 (0.77) | 3.92 (0.84) |
| g. My colleagues would not mind if I chose to walk over and talk to them (*iMail*) rather than sending them an eMail | 3.84 (0.78) | 3.87 (0.72) | 3.99 (0.64) | 4.11 (0.68) | 3.92 (0.69) | 4.07 (0.67) |
| h. My supervisor/s would not mind if I chose to walk over and talk to them (*iMail*) rather than sending them an eMail | 3.74 (0.92) | 3.71 (0.80) | 3.81 (0.90) | 3.87 (0.96) | 3.84 (0.78) | 3.93 (0.77) |
| Overall perceived organisational norms score | 3.54 (0.57) | 3.66 (0.52) | 3.60 (0.58) | 3.96 (0.60) | 3.66 (0.54) | 3.86 (0.60) |
| **Knowledge** |  |  |  |  |  |  |
| a. Sitting for most of the time at work does not impact on my health (reverse scored) | 2.13 (1.06) | 2.08 (1.07) | 2.26 (1.13) | 2.03 (1.06) | 1.95 (1.07) | 1.92 (1.02) |
| b. Sitting for most of the time at work is bad for my health | 3.79 (1.16) | 4.04 (0.91) | 3.95 (0.96) | 4.00 (1.06) | 4.09 (0.93) | 4.09 (1.06) |
| c. Any health impact of sitting for most of the time at work can be off-set by exercising at other times of the day (reverse scored) | 3.27 (0.92) | 3.00 (0.91) | 3.31 (1.00) | 2.58 (1.06) | 3.17 (0.93) | 2.66 (1.02) |
| d. It is beneficial for my health to stand up at least once every 30 minutes while I am at work | 4.11 (0.76) | 4.10 (0.80) | 4.23 (0.74) | 4.40 (0.70) | 4.39 (0.72) | 4.39 (0.77) |
| e. It is beneficial for my health if I am as active as possible throughout my working day (e.g. by using the stairs instead of the lift) | 4.22 (0.78) | 4.26 (0.75) | 4.26 (0.85) | 4.50 (0.61) | 4.33 (0.81) | 4.50 (0.72) |
| Overall knowledge score | 3.74 (0.56) | 3.86 (0.51) | 3.77 (0.56) | 4.06 (0.57) | 3.94 (0.57) | 4.08 (0.57) |

Note: For all questions and scales, minimum score=1; maximum score=5. Mean (standard deviation) are calculated with linearized variance estimation.

^a^ n=95 for barrier self-efficacy and perceived organisational social norms
