# Supplementary material for: Intervening to reduce workplace sitting: mediating role of social-cognitive constructs during a cluster randomised controlled trial
Source: Int J Behav Nutr Phys Act. 2017 Mar 6;14:27. doi: 10.1186/s12966-017-0483-1 (PMC5340005; doi:10.1186/s12966-017-0483-1)
Supplement: Additional file 2: — Effect of the SUV intervention on targeted social-cognitive constructs at 3 and 12 months – intention to treat analysis. (DOCX 15 kb) [file 12966_2017_483_MOESM2_ESM.docx]

Additional file 2: Effect of the SUV intervention on targeted social-cognitive constructs at three and 12 months – intention to treat analysis

|  |  | Mean change (SE)^a^ | | Intervention effect (95% CI) ^a b^ | p |
| --- | --- | --- | --- | --- | --- |
|  |  | Intervention n= 136 | Control  n=95 |  |  |
| Perceived behavioural control | 3 months | 0.81 (0.08) | 0.18 (0.08) | 0.69 (0.51, 0.87) | <0.001 |
|  | 12 months | 0.77 (0.14) | 0.17 (0.12) | 0.61 (0.24, 0.98) | 0.001 |
| Barrier self-efficacy | 3 months | 0.93 (0.08) | 0.13 (0.11) | 0.93 (0.67, 1.19) | <0.001 |
|  | 12 months | 0.72 (0.11) | 0.17 (0.17) | 0.66 (0.25, 1.06) | 0.002 |
| Perceived organisational norms | 3 months | 0.31 (0.05) | 0.07 (0.05) | 0.28 (0.11, 0.45) | 0.002 |
|  | 12 months | 0.20 (0.02) | 0.09 (0.14) | 0.09 (-0.32, 0.50) | 0.658 |
| Knowledge | 3 months | 0.19 (0.05) | 0.04 (0.07) | 0.22 (0.05, 0.38) | 0.013 |
|  | 12 months | 0.20 (0.08) | 0.20 (0.09) | 0.06 (-0.23, 0.35) | 0.684 |

Note: For each construct, minimum score = 1 and maximum score = 5. CI = confidence interval, ICC = intracluster correlation.

^a^ Missing data imputed by chained equations, m=30 imputations (largest fraction of missing information =0.29) ^b^ Assessed by mixed models, performed separately for short and long-term changes, with random intercept to correct for clustering, and adjusting as fixed effects for baseline values of the outcome, and other potential confounders measured at baseline: age, gender (male/female), workplace sitting time (min/8-h), Caucasian ethnicity (yes/no), current smoking (yes/no), body mass index (log-transformed), AQoL-8D physical superdomain score (log-transformed), AQoL-8D mental superdomain score (log-transformed), TV viewing time (log-transformed), job control category (high/low), weekly headaches (yes/no), musculoskeletal symptoms in the upper extremities (none/does not interfere with activities/interferes with activities).
