# Supplementary material for: Intervening to reduce workplace sitting: mediating role of social-cognitive constructs during a cluster randomised controlled trial
Source: Int J Behav Nutr Phys Act. 2017 Mar 6;14:27. doi: 10.1186/s12966-017-0483-1 (PMC5340005; doi:10.1186/s12966-017-0483-1)
Supplement: Additional file 3: — Worksite variation (ICCs) in changes in social-cognitive constructs at 3 and 12 months. (DOCX 14 kb) [file 12966_2017_483_MOESM3_ESM.docx]

Additional file 3: Worksite variation (ICCs) in changes in social-cognitive constructs at three and 12 months

|  | 3 months | | 12 months | |
| --- | --- | --- | --- | --- |
|  | ICC (95% CI) | p | ICC (95% CI) | p |
| Perceived behavioural control | ^a^ | >0.999 | 0.128 (0.029, 0.421) | 0.014 |
| Barrier self-efficacy | 0.007 (<0.001, 0.978) | 0.405 | 0.032 (0.001, 0.664) | 0.282 |
| Perceived organisational norms | 0.027 (0.001, 0.458) | 0.239 | 0.169 (0.042, 0.487) | 0.003 |
| Knowledge | ^a^ | >0.999 | 0.088 (0.013, 0.407) | 0.061 |

ICC = intracluster correlation coefficient; CI = confidence interval

^a^ ICC inestimably small <0.001
